# Therapeutic adherence and associated factors among high-risk patients with type 2 diabetes in primary healthcare: a cross-sectional study using real-world data from the Madrid Region

**DOI:** 10.3389/fendo.2026.1947294

**Published:** 2026-09-07

**Authors:** María de la Concepción Martín Trujillo, Ana Villimar Rodríguez, Andrés Gaspar Castillo Sanz, Jaime Barrio-Cortes

**Affiliations:** 1 Doctoral Program in Health Sciences, Faculty HM of Health Sciences, University Camilo José Cela (UCJC), Madrid, Spain; 2 Primary Care Pharmacy, Care Management of the Central Territorial Area, Madrid Health Service, Madrid, Spain; 3 Foundation for Biomedical Research of Hospital Niño Jesús (FIBHUNJ), Madrid, Spain; 4 HM Hospitales Research Institute, Madrid, Spain; 5 Foundation for Biosanitary Research and Innovation in Primary Health Care (FIIBAP), Madrid, Spain; 6 Research Network on Chronicity, Primary Health Care and Prevention and Health Promotion (RICAPPS), Carlos III Health Institute, Madrid, Spain

**Keywords:** clinical complexity, high-risk patients, multimorbidity, primary care, real-word data, therapeutic adherence, type 2 diabetes

## Abstract

**Background:**

Therapeutic adherence is a key component of chronic disease management, particularly in high-risk patients. Type 2 diabetes (T2DM) is common in this population; however, real-world evidence on therapeutic adherence and the factors associated with it among high-risk patients with T2DM remains limited in Spain and other regions.

**Objective:**

To evaluate therapeutic adherence and identify factors associated with nonadherence among high-risk patients with T2DM using real-world primary care data.

**Materials and methods:**

A cross-sectional study was conducted among adults with T2DM classified as high-risk chronic patients using primary healthcare electronic health records from the Madrid Region as of 30 April 2021. Sociodemographic, functional, clinical, lifestyle, pharmacological, and primary healthcare utilization variables were analyzed according to therapeutic adherence status. Multivariable logistic regression was performed to identify factors independently associated with therapeutic nonadherence.

**Results:**

Among 67,746 high-risk patients with T2DM, adherence information was available for 65,856 patients, of whom 45,669 (69.3%) were classified as nonadherent. Nonadherent patients had a higher prevalence of polypharmacy (62.1% vs. 58.7%) and greater healthcare utilization, with a higher mean number of primary care contacts during the first year (25.3 vs. 24.4). In the multivariable analysis, the independent factors associated with higher odds of nonadherence were older age (odds ratio (OR = 1.008), male sex (OR = 1.073), foreign status (OR = 1.157), Individual Health Card category 3 (annual income up to €18,000) (OR = 1.245), ischaemic heart disease (OR = 1.137), heart failure (OR = 1.072), COPD (OR = 1.571), asthma (OR = 1.622), depression (OR = 1.054), palliative care (OR = 1.352), smoking status (OR = 1.102), polypharmacy (OR = 1.345), and ≥20 primary care contacts (OR = 1.066).

**Conclusions:**

Therapeutic nonadherence is highly prevalent among high-risk patients with T2DM managed in routine primary care. Nonadherence was independently associated with treatment burden, specific patterns of clinical complexity, behavioral factors, and healthcare needs, with some associations showing modest effect sizes, highlighting the multifactorial nature of therapeutic adherence in this population. These findings support the integration of routine adherence assessment and individualized medication management strategies into primary care for patients with complex chronic conditions.

## 1 Introduction

Therapeutic adherence is a key component of chronic disease management, as it directly influences the effectiveness of interventions, clinical outcomes, and the prevention of long-term complications. It refers to the extent to which a person’s behavior aligns with agreed-upon health recommendations, including the sustained implementation of treatment and self-care behaviors over time. Poor therapeutic adherence remains a major public health challenge worldwide and is associated with increased morbidity, mortality, healthcare utilization, and healthcare costs across a wide range of chronic diseases (1).

Adherence-related challenges are especially pronounced among high-risk chronic patients, who frequently present multimorbidity and complex care needs. This multidimensional perspective is particularly relevant in primary care, where these patients are managed longitudinally and identified through morbidity-based stratification systems such as adjusted morbidity groups (AMG) (2, 3). In this setting, healthcare professionals play a central role in coordinating care, reviewing medication, and supporting long-term therapeutic adherence.

In this population, T2DM is among the most prevalent chronic conditions (4, 5). It frequently coexists with other chronic diseases, contributing to greater multimorbidity, increased healthcare utilization, and more complex care trajectories, all of which may hinder the achievement of therapeutic goals (4, 5). As the prevalence of T2DM continues to increase with population ageing, optimizing therapeutic adherence has become an important priority for improving long-term outcomes and reducing the burden on healthcare systems.

In patients with T2DM, therapeutic adherence is particularly relevant because of the chronic nature of the disease, the need for long-term pharmacological and lifestyle interventions, and the frequent presence of comorbidities that increase the complexity of self-management. Current recommendations from the American Diabetes Association (ADA) and the European Association for the Study of Diabetes (EASD) emphasize person-centered care and comprehensive assessment integrating biomedical, psychological, functional, and social dimensions, given their influence on self-management capacity and clinical outcomes. Moreover, suboptimal therapeutic adherence has been consistently associated with poorer glycaemic control, increased risk of diabetes-related complications, higher rates of hospitalization, and higher healthcare costs. Consequently, improving adherence has become a key objective in the management of patients with T2DM, particularly those with complex care needs (1, 6–8).

Recent research on multimorbidity has also highlighted treatment burden as a key determinant of patient experience, which may negatively affect adherence and continuity of care (3). More complex therapeutic regimens increase treatment burden and self-management demands, potentially compromising therapeutic adherence (7, 8). In addition, adherence is influenced by multiple interacting factors, including functional status, self-management capacity, social determinants, and patterns of healthcare utilization (9–11). Although several determinants of therapeutic adherence have been described in patients with T2DM, previous studies have mainly examined sociodemographic, clinical, behavioral, and treatment-related factors in broader populations of patients with T2DM, including those with multimorbidity (10, 12). Recent real-world studies have also examined adherence trajectories and associated factors in patients with T2DM, including clinical outcomes, diabetes-related knowledge and self-management factors (13, 14). However, less is known about how these factors interact among patients identified as high-risk through morbidity-based risk stratification systems, particularly in the context of routine primary care (15). Evidence integrating these dimensions to understand therapeutic adherence within this specific risk-stratified population and care setting remains limited.

This study was conducted within the European Horizon 2020 Innovative Medicines Initiative BEAMER project (BEhavioral and Adherence Model for improving quality, health outcomes, and cost-Effectiveness of healthcaRe), which aims to improve the understanding of therapeutic adherence and support the development of predictive and targeted care strategies across healthcare settings (16). Within this framework, examining therapeutic adherence and the factors associated with it in high-risk patients with T2DM may provide clinically relevant evidence to better understand adherence-related challenges in routine primary care. Therefore, this study aimed to analyze therapeutic adherence and identify factors independently associated with therapeutic nonadherence among high-risk patients with T2DM managed in a real-world primary care setting.

## 2 Materials and methods

### 2.1 Study design

A cross-sectional descriptive observational study was conducted.

### 2.2 Study population and participant selection

The study was conducted in the Community of Madrid (Spain) using routine clinical practice data from primary care (17, 18). The Madrid Health Service (SERMAS) primary care electronic health record routinely identifies patients with chronic conditions and stratifies them according to morbidity burden and clinical complexity using the AMG stratification tool system. Patients are classified into four risk categories: high, medium, low, or without risk. High-risk patients are those above the 95th percentile of the AMG complexity score, representing patients with the greatest clinical complexity and healthcare needs, following the population risk stratification framework of the Kaiser Permanente pyramid and the King’s Fund model (2).

All adults (≥18 years) classified as high-risk chronic patients according to the AMG and recorded in the primary care electronic record of the SERMAS as of April 30, 2021, were considered for inclusion. For the present study, adults with T2DM who met these criteria were included. No sampling or additional selection of a subset of eligible patients was performed; the study included the entire population of high-risk patients with T2DM identified through this routine risk-stratification process, resulting in a study population of 67,746 high-risk chronic adults with T2DM. Nonchronic and chronic patients categorized as medium-risk, low-risk, or without risk were excluded (19).

The study population selection process is summarized in **Supplementary Figure 1**, showing the identification of the source population, AMG-based risk stratification, selection of high-risk patients with T2DM, and the availability of therapeutic adherence information.

### 2.3 Data collection

Secondary data were extracted from primary healthcare electronic health records and pharmacy dispensing records from the SERMAS. The data were extracted and anonymized by the Technical Directorate of Information Systems of Primary Care Management, while pharmaceutical data were provided by the Directorate General of Economic-Financial Management and Pharmacy of SERMAS. The electronic health records provided sociodemographic, functional, clinical, lifestyle-related, and primary healthcare utilization data. Pharmacy dispensing records were used to obtain information on prescribed and dispensed medications and to assess therapeutic adherence.

All variables were routinely recorded by primary healthcare professionals according to standardized clinical documentation protocols. For variables recorded longitudinally, the most recent value available at the time of data extraction (30 April 2021) was considered the baseline assessment. Primary healthcare utilization and pharmacy dispensing data were assessed over the subsequent 12 months (30 April 2021 to 30 April 2022).

### 2.4 Variables

Therapeutic adherence was the primary outcome. It was assessed using the proportion of days covered (PDC), calculated from pharmacy dispensing records over the subsequent year for baseline chronic medications belonging to the main therapeutic groups considered in the study: alimentary tract and metabolism (ATC A), blood and blood-forming organs (ATC B), cardiovascular system (ATC C), nervous system (ATC N) and respiratory system (ATC R). For each medication, coverage was estimated assuming the minimum recommended daily dose according to the Pharmacy Quality Alliance (PQA) consensus document drug information sheet. The PDC was calculated as the proportion of days during the observation period for which medication coverage was available. A PDC ≥ 80% was considered indicative of adherence, whereas a PDC < 80% was considered nonadherence, consistent with previous studies supporting this threshold as a commonly used indicator of adequate medication adherence. Although PDC is an indirect measure that does not capture actual medication intake, it is widely used in large-scale pharmacoepidemiological studies because of its practicality, reproducibility, and validity as a proxy for medication adherence (20–25).

The explanatory variables were grouped into six domains:

1. Sociodemographic variables: age, sex, foreign status, continent of birth, socioeconomic status measured using the deprivation index, and Individual Health Card (IHC) category.
2. Functional variables: functional status, living alone, homebound status or immobility status, presence and type of primary caregiver, and palliative care.
3. Clinical variables: AMG complexity index and AMG complexity quartiles, number of chronic diseases, multimorbidity, chronic disease clusters, and specific chronic diseases. The AMG tool, integrated into primary healthcare electronic health records, provides a morbidity-based measure of clinical complexity.
4. Lifestyle-related variables: tobacco use, alcohol use, illicit drug abuse, medication abuse, physical exercise, adherence to the Mediterranean diet, regular stress, normal sleep habits, and risk of sexually transmitted infections.
5. Pharmacological variables: number of medications, polypharmacy, anatomical therapeutic chemical (ATC) medication groups, medication-related concerns, adverse drug reactions, and treatment-related complications.
6. Primary care utilization variables: total number of primary care contacts, type of healthcare professional contacted, and mode of contact.

All the variables are defined in **Supplementary Table 1**.

### 2.5 Statistical analysis

Patients were compared according to therapeutic adherence status. Categorical variables were analyzed using the chi-square test or Fisher’s exact test, as appropriate. Continuous variables were compared using parametric or nonparametric tests according to their distribution.

Patients with unavailable therapeutic adherence data were excluded from the comparative analyses. For variables that were not recorded or were recorded as unknown, percentages were calculated using the available data for each variable unless otherwise specified.

A multivariable binary logistic regression model was constructed to identify factors independently associated with therapeutic nonadherence among high-risk patients with T2DM. Variables showing statistical significance in the bivariate analyses and/or considered clinically relevant according to previous evidence were included in the model. A forced-entry (Enter) approach was used, whereby all selected variables were entered simultaneously to estimate the independent association of each factor while adjusting for the potential confounding effects of the others. Variable selection was guided by clinical relevance and previous evidence, with the principle of parsimony applied to retain only clinically relevant covariates and those supported by the literature. Potential multicollinearity among the variables included in the model was assessed using the variance inflation factor. Adjusted odds ratios (OR) with 95% confidence intervals (CI) were estimated.

Statistical significance was established at *p* < 0.05. All analyses were performed using IBM SPSS Statistics version 30.0 (IBM Corp., Armonk, NY, USA).

## 3 Results

The final study population included 67,746 high-risk patients with T2DM. Information concerning therapeutic adherence was available for 65,856 patients, of whom 45,669 (69.3%) were classified as nonadherent and 20,187 (30.7%) as adherent. Therapeutic adherence could not be determined for the remaining 1,890 patients. Accordingly, descriptive analyses and comparisons according to adherence status were based on the 65,856 patients with available adherence information. The study population selection process is shown in **Supplementary Figure 1**.

### 3.1 Sociodemographic characteristics

Age and sex distributions were comparable between nonadherent and adherent patients. The proportion of patients aged ≥ 80 years was similar between groups (45.7% vs. 46.3%), and women accounted for approximately half of the study population (49.5% vs. 49.4%). Similarly, no significant differences were observed according to the continent of birth or deprivation index. Foreign-born individuals were more common among nonadherent patients than among adherent patients (2.3% vs. 2.0%). The distribution of IHC categories also differed between groups, although the absolute differences were small. IHC 002 (pensioners) was the most common category in both groups (63.3% vs. 63.8%), whereas IHC 003 (active citizens with annual income ≤€18,000) was more frequent among nonadherent patients (4.6% vs. 3.8%) and IHC 001 (exempt users, non-contributory pensioners, and long-term unemployed) among adherent patients (30.1% vs 30.6%) (**Table 1**).

**Table 1:** Sociodemographic characteristics of high-risk patients with T2DM overall and by therapeutic adherence status.

Sociodemographic characteristics n (%)	Patients with T2DM n= 65,856 (100)	Nonadherent n= 45,669 (69.3)	Adherent n= 20,187 (30.7)	p value
Age				
Mean (SD)	77.5 (10.3)	77.4 (10.3)	77.6 (10.4)	0.560
Median (IQR)	79 (14)	78 (14)	79 (14)	
Age ≥80 years	30,242 (45.9)	20,889 (45.7)	9,353 (46.3)	0.160
Sex				
Male	33,284 (50.5)	23,073 (50.5)	10,211 (50.6)	0.887
Female	32,572 (49.5)	22,596 (49.5)	9,976 (49.4)	
Foreign status				
Yes	1,460 (2.2)	1,060 (2.3)	400 (2.0)	0.006
No	64,396 (97.8)	44,609 (97.7)	19,787 (98.0)	
Continent of birth				
Africa	659 (1.0)	480 (1.1)	179 (0.9)	0.202
America	2,048 (3.1)	1,449 (3.2)	599 (3.0)	
Asia	240 (0.4)	160 (0.4)	80 (0.4)	
Europe	62,902 (95.5)	43,575 (95.4)	19,327 (95.7)	
Oceania	5 (0.0)	4 (0.0)	1 (0.0)	
Deprivation index				
Mean (SD)	0.1 (0.9)	0.1 (0.9)	0.1 (0.9)	0.624
Median (IQR)	0.2 (1.3)	0.2 (1.3)	0.2 (1.3)	
1st quartile (Q1)	16,203 (24.6)	11,240 (24.6)	4,963 (24.6)	0.672
2nd quartile (Q2)	16,777 (25.5)	11,663 (25.5)	5,114 (25.3)	
3rd quartile (Q3)	17,007 (25.8)	11,822 (25.9)	5,185 (25.7)	
4th quartile (Q4)	15,869 (24.1)	10,944 (24.0)	4,925 (24.4)	
IHC categories				
IHC 001	19,771 (30.2)	13,640 (30.1)	6,131 (30.6)	<0.001
IHC 002	41,484 (63.5)	28,696 (63.3)	12,788 (63.8)	
IHC 003	2,846 (4.4)	2,088 (4.6)	758 (3.8)	
IHC 004	1,004 (1.5)	697 (1.5)	307 (1.5)	
IHC 005	269 (0.4)	197 (0.4)	72 (0.4)	
IHC 006	2 (0.0)	1 (0.0)	1 (0.0)	
Other IHC categories	480 (0. 7)	350 (0.8)	130 (0.6)	

Individual Health Card (IHC) income category; IHC 1, exempt users, non-contributory pensioners, and long-term unemployed; IHC 2, pensioners (10% co-payment); IHC 3, active citizens with annual income ≤€18,000 (40% co-payment); IHC 4, active citizens with annual income €18,001– €100,000 (50% co-payment); IHC 5, citizens with annual income >€100,000 (60% co-payment); IHC 6, members of mutual insurance schemes; IQR, interquartile range; Q, Quartile; SD, standard deviation; T2DM, Type 2 diabetes mellitus. Patients with unavailable therapeutic adherence data were excluded from the analyses (n = 1,890). Continent of birth was unclassified for 2 patients.

### 3.2 Functional characteristics

The proportion of independent patients was similar between nonadherent and adherent patients (78.1% vs. 78.8%), as was homebound status or immobility (12.2% vs. 12.8%), Among patients with available information, living alone did not differ significantly between groups, although this variable had a high proportion of missing data. The distribution of primary caregiver categories differed significantly between the adherence groups, although the absolute differences were small. Having a recorded primary caregiver was slightly more common among nonadherent patients (17.9% vs. 17.3%). regarding caregiver type, offspring and partners were slightly more common in the nonadherent group (7.0% vs. 6.4% and 5.0% vs. 4.6%, respectively), whereas in-home help was slightly more frequent among adherent patients (1.7% vs. 1.8%). Palliative care was more frequent among nonadherent patients than among adherent patients (1.5% vs. 1.1%) (**Table 2**).

**Table 2:** Functional characteristics of high-risk patients with T2DM overall and by therapeutic adherence status.

Functional characteristics n (%)	Patients with T2DM n= 65,856 (100)	Nonadherent n= 45,669 (69.3)	Adherent n= 20,187 (30.7)	p value
Functional status				
Independent	51,598 (78.3)	35,690 (78.1)	15,908 (78.8)	0.060
Dependent	14,258 (21.7)	9,979 (21.9)	4,279 (21.2)	
Living situation				
Living alone	3,183 (4.8)	2,252 (4.9)	931 (4.6)	0.468
Immobilized	8,166 (12.4)	5,592 (12.2)	2,574 (12.8)	0.069
Needs of care				
Primary caregiver	11,649 (17.7)	8,166 (17.9)	3,483 (17.3)	0.052
Offspring	4,485 (6.8)	3,185 (7.0)	1,300 (6.4)	0.021
Partner	3,201 (4.9)	2,264 (5.0)	937 (4.6)	
In-home help	1,130 (1.7)	776 (1.7)	354 (1.8)	
Parents	35 (0.1)	26 (0.1)	9 (0.0)	
Other relatives	636 (1.0)	455 (1.0)	181 (0.9)	
Others	2,162 (3.3)	1,460 (3.2)	702 (3.5)	
Palliative care	929 (1.4)	705 (1.5)	224 (1.1)	<0.001

T2DM, Type 2 diabetes mellitus. Patients with unavailable therapeutic adherence data were excluded from the analyses (n = 1,890).

### 3.3 Clinical characteristics

Patients in the nonadherent group showed slightly greater clinical complexity than adherent patients did. The highest AMG complexity quartile (Q4) was more frequent among nonadherent patients (30.2% vs. 27.4%), whereas the lowest quartile (Q1) was slightly more frequent among adherent patients (21.3% vs. 22.8%). Similarly, nonadherent patients had a slightly greater mean number of chronic diseases (8.1% vs. 7.8%). Multimorbidity and endocrinological and metabolic diseases were present in all patients by definition. Cardiovascular diseases were highly prevalent in both groups (98.7%), with no significant differences. Mental and behavioral disorders were slightly more frequent among nonadherent patients (31.1% vs. 30.2%), whereas neurological diseases were slightly more frequent among adherent patients (51.0% vs 51.8%). No significant differences were observed for oncological diseases (**Table 3**). With respect to specific chronic diseases, several conditions, including ischaemic heart disease, heart failure, cardiomyopathy, cardiopulmonary disease, obesity, hyperlipidaemia, chronic obstructive pulmonary disease (COPD), asthma, depression, and alcohol abuse disorder were more frequent among nonadherent patients. Conversely, dementia, Parkinson’s disease, epilepsy, paralysis, bipolar disorder, psychotic disorder, anemia, and colon cancer were more frequent among adherent patients (**Supplementary Table 2**).

**Table 3:** Clinical characteristics of high-risk patients with T2DM overall and by therapeutic adherence status.

Clinical characteristics n (%)	Patients with T2DM n= 65,856 (100)	Nonadherent n= 45,669 (69.3)	Adherent n= 20,187 (30.7)	p value
Complexity index by adjusted morbidity groups				
1st quartile (Q1)	14,310 (21.7)	9,713 (21.3)	4,597 (22.8)	<0.001
2nd quartile (Q2)	15,404 (23.4)	10,531 (23.1)	4,873 (24.1)	
3rd quartile (Q3)	16,817 (25.5)	11,627 (25.5)	5,190 (25.7)	
4th quartile (Q4)	19,325 (29.3)	13,798 (30.2)	5,527 (27.4)	
Number of chronic diseases				
Mean (SD)	8.0 (2.2)	8.1 (2.2)	7.8 (2.1)	<0.001
Median (IQR)	8 (2)	8 (2)	8 (3)	
Multimorbidity (≥2)	65,856 (100.0)	45,669 (100.0)	20,187 (100.0)	--
Disease clusters				
Cardiovascular	64,996 (98.7)	45,068 (98.7)	19,928 (98.7)	0.731
Endocrinological and metabolic	65,856 (100.0)	45,669 (100.0)	20,187 (100.0)	--
Mental and behavioral	20,291 (30.8)	14,192 (31.1)	6,099 (30.2)	0.027
Neurological diseases	33,736 (51.2)	23,279 (51.0)	10,457 (51.8)	0.050
Oncological	8,211 (12.5)	5,629 (12.3)	2,582 (12.8)	0.096

AMG, adjusted morbidity group; IQR, interquartile range; Q, quartile; SD, standard deviation; T2DM, type 2 diabetes mellitus. Patients with unavailable therapeutic adherence data were excluded from the analyses (n = 1,890).

### 3.4 Lifestyle-related characteristics

Tobacco use and alcohol abuse showed small but statistically significant differences between groups. Both were more frequent among nonadherent patients, with rates of 7.2% vs. 6.0% for tobacco use and 5.0% vs. 4.6% for alcohol abuse. No significant differences were observed for illicit drug abuse or medication abuse. Among patients with recorded data, adherent patients were slightly more likely to report regular physical exercise than nonadherent patients (53.5% vs. 55.0%). Similarly, reporting Mediterranean diet was slightly more frequent among adherent patients (92.3% vs. 91.4%). Although these differences were statistically significant, the absolute differences were small. Regular stress, normal sleep habits, and the risk of sexually transmitted infections were poorly recorded, with a high proportion of unrecorded values, limiting meaningful comparisons between groups (**Table 4***)*.

**Table 4:** Lifestyle-related characteristics of high-risk patients with T2DM overall and by therapeutic adherence status.

Lifestyle characteristics n (%)	Patients with T2DM n= 65,856 (100)	Nonadherent n= 45,669 (69.3)	Adherent n= 20,187 (30.7)	p value
Substance use behaviors				
Tobacco use	4,486 (6.8)	3,284 (7.2)	1,202 (6.0)	<0.001
Alcohol abuse	3,234 (4.9)	2,300 (5.0)	934 (4.6)	0.025
Illicit drug abuse	369 (0.6)	270 (0.6)	99 (0.5)	0.110
Medication abuse	155 (0.2)	114 (0.2)	41 (0.2)	0.256
Physical exercise				
Yes	16,977 (25.8)	11,688 (25.6)	5,289 (26.2)	0.014
No	14,481 (46.0)	10,156 (46.5)	4,325 (45.0)	
Unknown	34,398			
Mediterranean diet				
Yes	27,467 (41.7)	18,940 (91.4)	8,527 (92.3)	0.005
No	2,502 (8.3)	1,793 (8.6)	709 (7.7)	
Unknown	35,887			
Regular stress				
Yes	43 (25.6)	33 (29.2)	10 (18.2)	0.136
No	125 (74.4)	80 (70.8)	45 (81.8)	
Unknown	65,688			
Normal sleep habits				
Yes	2 (66.7)	1 (100.0)	1 (50.0)	1.000
No	1 (33.3)	0 (0.0)	1 (50.0)	
Unknown	5,853			
Risk of sexually transmitted infections				
Yes	18 (1.4)	11 (1.1)	7 (1.9)	0.287
No	1,309 (98.6)	956 (98.9)	353 (98.1)	
Unknown	64,529			

T2DM, Type 2 diabetes mellitus. Patients with unavailable therapeutic adherence data were excluded from the analyses (n = 1,890). For lifestyle variables with an unknown category, percentages in the descriptive analysis and P values in the bivariate analysis were calculated only among patients with recorded Yes/No responses.

### 3.5 Pharmacological characteristics

Compared with adherent patients, nonadherent patients had a greater pharmacological burden. They had a higher mean number of medications (10.8 vs. 8.9), and polypharmacy was also more frequent (62.1% vs. 58.7%). With respect to medication classes, the use of ATC group A medications was more frequent among nonadherent patients than among adherent patients (98.0% vs. 95.5%). A statistically significant difference was also observed for ATC group N medications (94.4% vs. 94.0%), although the absolute difference was minimal. No significant differences were found for ATC groups B, C, or R. Medication-related concerns were similarly distributed between groups. However, adverse drug reactions were slightly more frequent among nonadherent patients than among adherent patients (9.4% vs. 8.8%), whereas treatment-related complications did not differ significantly between groups (**Table 5**).

**Table 5:** Pharmacological characteristics of high-risk patients with T2DM overall and by therapeutic adherence status.

Pharmacological characteristics n (%)	Patients with T2DM n= 65,856 (100)	Nonadherent n= 45,669 (69.3)	Adherent n= 20,187 (30.7)	p value
Number of medications				
Mean (SD)	10.2 (3.5)	10.8 (3.6)	8.9 (3.0)	<0.001
Median (IQR)	10 (4)	11 (5)	9 (4)	
Polypharmacy (≥5)	40,201 (61.0)	28,345 (62.1)	11,856 (58.7)	<0.001
ATC medication groups				
ATC group A medication use	64,054 (97.3)	44,776 (98.0)	19,278 (95.5)	<0.001
ATC group B medication use	49,653 (75.4)	34,422 (75.4)	15,231 (75.4)	0.833
ATC group C medication use	60,871 (92.4)	42,196 (92.4)	18,675 (92.5)	0.608
ATC group N medication use	62,099 (94.3)	43,129 (94.4)	18,970 (94.0)	0.017
ATC group R medication use	25,365 (38.5)	17,658 (38.7)	7,707 (38.2)	0.236
Medication-related concerns and complications				
Concern about medication	21,584 (32.8)	14,989 (32.8)	6,595 (32.7)	0.703
Adverse drug reactions	6,061 (9.2)	4,292 (9.4)	1,769 (8.8)	0.009
Treatment complications	2,610 (4.0)	1,816 (4.0)	794 (3.9)	0.793

ATC, anatomical therapeutic chemical; IQR, interquartile range; SD, standard deviation; T2DM, type 2 diabetes mellitus. Patients with unavailable therapeutic adherence data were excluded from the analyses (n = 1,890).

### 3.6 Primary care utilization

Primary care utilization according to therapeutic adherence status is presented in **Table 6**. Overall, compared with adherent patients, nonadherent patients had greater use of primary care services. They recorded a higher mean number of total primary care contacts during the first year (25.3 vs. 24.4), together with more contacts with family physicians (11.3 vs. 10.5). Nonadherent patients also had more in-person consultations (19.4 vs. 18.4) and telephone consultations (12.1 vs. 11.5). All these differences were statistically significant. In contrast, no significant differences were observed in contacts with primary care nurses and primary care pharmacists, as well as home visits did not differ significantly between groups.

**Table 6:** Utilization of primary care services among high-risk patients with T2DM overall and by therapeutic adherence status.

Primary care utilization n (%)	Patients with T2DM n= 65,856 (100)	Nonadherent n=45,669 (69.3)	Adherent n=20,187 (30.7)	p value
Total contacts				
Mean (SD)	25.05 (18.5)	25.32 (18.6)	24.42 (18.4)	<0.001
Median (IQR)	21 (19)	21 (20)	21 (20)	
Main professionals contacted				
Family medicine physician				
Mean (SD)	11.0 (8.4)	11.3 (8.6)	10.5 (8.1)	<0.001
Median (IQR)	9 (10)	10 (10)	9 (9)	
Primary care nurse				
Mean (SD)	7.2 (10.9)	7.2 (10.9)	7.2 (11.2)	0.744
Median (IQR)	4 (9)	4 (9)	4 (9)	
Primary care pharmacist				
Mean (SD)	0.04 (0.3)	0.04 (0. 3)	0.04 (0.3)	1
Median (IQR)	0 (0)	0 (0)	0 (0)	
Mode of contact				
In-person				
Mean (SD)	19.1 (15.8)	19.4 (15.9)	18.4 (15.4)	<0.001
Median (IQR)	15 (17)	16 (18)	15 (16)	
Telephone				
Mean (SD)	11.9 (11.2)	12.1(11.4)	11.5 (10.7)	<0.001
Median (IQR)	9 (12)	9 (12)	9 (12)	
Home visits				
Mean (SD)	1.7 (7.1)	1.7 (6.9)	1.8 (7.7)	0.461
Median (IQR)	0 (0)	0 (0)	0 (0)	

SD, standard deviation; IQR, interquartile range; T2DM, type 2 diabetes mellitus. Patients with unavailable therapeutic adherence data were excluded from the analyses (n = 1,890).

### 3.7 Multivariable analysis

Multivariable logistic regression analysis revealed several factors independently associated with therapeutic nonadherence (**Table 7**). Older age (OR = 1.008), male sex (OR = 1.073), foreign status (OR = 1.157), belonging to the IHC 003 category (active citizens with an annual income up to €18,000; OR = 1.245), and receiving palliative care (OR = 1.352) were independently associated with higher odds of nonadherence. In terms of clinical characteristics, ischaemic heart disease (OR = 1.137), heart failure (OR = 1.072), COPD (OR = 1.571), asthma (OR = 1.622), and depression (OR = 1.054) were independently associated with higher odds of nonadherence. In contrast, dementia (OR = 0.874) and anemia (OR = 0.903) were associated with lower odds of nonadherence. Current smoking (OR = 1.102), polypharmacy (OR = 1.345), and having ≥ 20 primary care contacts/year (OR = 1.066) were also independently associated with higher odds of therapeutic nonadherence.

**Table 7:** Multivariable logistic regression analysis of factors independently associated with therapeutic nonadherence among high-risk patients with T2DM.

Variable	OR	95% CI	*P* value
Age (per year)	1.008	1.006–1.011	<0.001
Male sex	1.073	1.034–1.114	<0.001
Foreign status	1.157	1.024–1.306	0.019
Palliative care	1.352	1.160–1.576	<0.001
IHC 003	1.245	1.135–1.365	<0.001
Ischaemic heart disease	1.137	1.094–1.181	<0.001
Heart failure	1.072	1.030–1.117	0.001
COPD	1.571	1.490–1.658	<0.001
Asthma	1.622	1.536–1.712	<0.001
Depression	1.054	1.013–1.096	0.009
Dementia	0.874	0.828–0.924	<0.001
Anemia	0.903	0.871–0.935	<0.001
Current smoker	1.102	1.026–1.184	0.007
Polypharmacy	1.345	1.280–1.414	<0.001
≥20 primary care contacts/year	1.066	1.031–1.103	<0.001

R2 de Nagelkerke= 0.13. CI, confidence interval; COPD, chronic obstructive pulmonary disease; OR, odds ratio; IHC 003, Individual Health Card income category corresponding to active citizens with an annual income ≤€18,000. Patients with unavailable therapeutic adherence data were excluded from the analyses (n = 1,890).

## 4 Discussion

### 4.1 Main findings

In this large real-world cohort of high-risk patients with T2DM managed in primary care, therapeutic nonadherence was frequent, affecting 69.3% of patients for whom adherence information was available. These findings highlight the magnitude of nonadherence in a population characterized by advanced age, universal multimorbidity, high clinical complexity, and substantial pharmacological burden.

Sociodemographic differences between adherent and nonadherent patients were limited in the descriptive analysis. Age and sex distributions were comparable between groups, and no relevant differences were observed according to the deprivation index or continent of birth. Foreign status and IHC category showed statistically significant differences, although the absolute differences were small. In particular, the IHC 003 category, corresponding to active citizens with an annual income ≤ €18,000, was more frequent among nonadherent patients. However, no differences were observed according to the deprivation index, suggesting that the relationship between socioeconomic-related factors and therapeutic adherence is complex and may not be fully captured by available socioeconomic indicators.

The functional characteristics were broadly comparable between the groups. Differences in dependency, homebound status, living arrangements, and caregiver distribution were small. However, palliative care was more frequent among nonadherent patients and remained independently associated with nonadherence, suggesting that advanced clinical needs, competing therapeutic priorities, and complex care situations may influence medication management.

Clinical complexity showed a clearer association with nonadherence. A greater proportion of nonadherent patients were in the highest AMG complexity quartile and had slightly more chronic diseases within this universally multimorbid population. Several specific conditions, particularly ischaemic heart disease, heart failure, COPD, asthma, and depression, were more frequent found among nonadherent patients and remained independently associated with nonadherence after adjustment. The association with depression is clinically relevant, as mental health conditions may negatively affect self-management, treatment engagement, and the ability to maintain complex medication regimens. Conversely, dementia and anemia were associated with lower odds of nonadherence in the adjusted analysis. Although these findings should be interpreted cautiously, the association observed for dementia may partly reflect greater medication supervision by caregivers or healthcare professionals. However, because PDC reflects medication dispensing rather than actual medication intake, higher PDC values among patients with dementia—particularly those who are supervised or institutionalized—may partly reflect medications being dispensed on their behalf and therefore may not necessarily indicate better medication-taking behavior. The association observed for anemia may similarly reflect differences in healthcare monitoring or underlying patient profiles rather than a direct effect on adherence.

Lifestyle-related characteristics differed only slightly between groups. Tobacco and alcohol use were slightly more common among nonadherent patients, whereas physical exercise and Mediterranean diet adherence were slightly more common among adherent patients among those with available information. Although these differences were modest and affected by missing data, smoking status remained independently associated with higher odds of nonadherence, suggesting that behavioral factors may reflect broader difficulties in chronic disease management.

The most consistent differences were observed in the pharmacological domain. Nonadherent patients had a higher treatment burden, with a higher incidence of polypharmacy. Polypharmacy remained independently associated with nonadherence after adjustment, highlighting the relevance of medication complexity in this population, where multiple treatments are frequently needed because of the coexistence of several chronic conditions.

Primary care utilization also differed between groups. Nonadherent patients had a greater number of total primary care contacts, including more family physician consultations and more in-person and telephone contacts. The association between higher healthcare utilization and nonadherence likely reflects greater healthcare needs and clinical complexity among these patients rather than a direct negative effect of healthcare contact.

Taken together, these findings suggest that therapeutic nonadherence among high-risk patients with T2DM is closely related to the interactions among patient characteristics, specific comorbidity patterns, treatment burden, behavioral factors, and healthcare needs. Multivariable analysis further supported the relevance of these factors beyond crude differences between groups.

### 4.2 Comparison with other studies

The high prevalence of therapeutic nonadherence observed in this cohort is consistent with previous evidence indicating that adherence remains a major challenge in T2DM management. Systematic reviews and meta-analyses have reported substantial variability in adherence estimates, largely reflecting differences in study populations, healthcare settings, adherence definitions, and measurement methods (9, 11). The present study extends previous evidence by focusing specifically on high-risk patients with T2DM managed in primary care, a population characterized by advanced age, universal multimorbidity, and high treatment complexity that remains underrepresented in adherence research.

Previous studies have identified sociodemographic characteristics as potential determinants of therapeutic adherence, although their effects are often inconsistent across populations (10, 11). In our cohort, sociodemographic differences were generally limited in the descriptive analysis. However, older age, male sex, foreign status, and the IHC 003 category were independently associated with nonadherence after adjustment, suggesting that demographic and social factors may contribute to adherence variability, although their influence is likely intertwined with broader differences in clinical complexity, treatment burden, and healthcare needs. Socioeconomic-related variables should be interpreted with caution, as available indicators may not fully capture the multidimensional nature of social determinants of health.

Functional status and social support have also been proposed as relevant determinants of medication adherence, particularly among older adults with multimorbidity. Previous studies have suggested that dependency, cognitive impairment, caregiver availability, and living circumstances may influence medication management (10, 11). In our study, functional dependency, homebound status, living arrangements, and caregiver characteristics showed limited differences between adherence groups. Nevertheless, palliative care was independently associated with nonadherence, potentially reflecting the complexity of medication management in patients with advanced disease, competing therapeutic priorities, and increased care needs.

The relationship between multimorbidity and therapeutic adherence has been extensively described. Patients with T2DM frequently require the management of multiple chronic conditions, which may increase treatment complexity and reduce the ability to maintain long-term adherence (9–11, 26). Consistent with previous findings, nonadherent patients in our cohort had greater clinical complexity and a higher prevalence of selected cardiovascular, respiratory, and mental health conditions. COPD, asthma, ischaemic heart disease, heart failure, and depression remained independently associated with nonadherence after adjustment, suggesting that specific comorbidity profiles may contribute beyond overall complexity. The association between depression and therapeutic nonadherence is particularly relevant, as depressive symptoms may negatively influence motivation, self-care behaviors, treatment engagement, and the ability to manage complex medication regimens. Conversely, global measures of complexity and the total number of chronic diseases were not retained in the final model, likely because of their close relationship with individual comorbidities and potential overlap of information.

Lifestyle-related factors have also been associated with adherence behaviors in patients with T2DM. Previous studies have reported that smoking, alcohol consumption, and other behavioral factors may negatively influence diabetes self-management and medication-taking behaviors (10, 11). In our cohort, differences in lifestyle characteristics were generally small, although tobacco and alcohol use were more frequent among nonadherent patients. Current smoking status remained independently associated with nonadherence, suggesting that behavioral factors may act as markers of broader difficulties in chronic disease management rather than as isolated determinants of medication-taking behavior.

Medication burden is among the most consistently reported determinants of adherence in patients with chronic diseases. Previous studies have shown that increasing numbers of medications, complex dosing schedules, and polypharmacy may negatively affect adherence, particularly among older adults with multimorbidity (26, 27). In agreement with this evidence, nonadherent patients in our cohort had a higher incidence of polypharmacy, which remained independently associated with nonadherence after adjustment. These findings reinforce the importance of balancing appropriate treatment intensity with avoidable therapeutic complexity. In high-risk patients with T2DM, medication review and treatment optimization are essential components of adherence management.

Healthcare utilization has increasingly been considered an indicator of clinical complexity and healthcare needs among patients with multimorbidity. Previous studies have suggested that frequent healthcare contacts may reflect greater disease burden and opportunities for adherence assessment rather than representing a direct determinant of poor adherence. In our cohort, nonadherent patients had higher primary care utilization, and having ≥ 20 primary care contacts remained independently associated with nonadherence after adjustment. This association likely reflects greater healthcare needs and clinical complexity among patients experiencing adherence difficulties rather than a negative effect of healthcare contact itself. Regarding the type of healthcare professional involved, contacts with family physicians were more frequent among nonadherent patients, whereas contacts with primary care nurses were slightly more frequent among adherent patients; however, the latter difference was not statistically significant.

Although similar patterns have been reported in previous studies (9, 28–33), suggesting that nurse-led activities focused on education, self-management support, treatment monitoring, and the reinforcement of adherence behaviors could play a relevant role in supporting long-term medication management in patients with T2DM, our cross-sectional findings did not demonstrate a significant association between nurse contacts and adherence. This finding should therefore be interpreted cautiously and considered exploratory. Further longitudinal studies are needed to clarify whether the type and intensity of healthcare professional contact influence medication adherence over time.

Multidisciplinary primary care models, integrating complementary roles for physicians, nurses, and pharmacists, may support long-term diabetes management (33). In Spain, the INTENSE study reported adherence challenges among patients with T2DM managed in primary care centers, reinforcing the relevance of evaluating adherence in real-world primary care populations (34). More recent Spanish evidence has also highlighted the association between therapeutic adherence and diabetes outcomes, including glycaemic control (35).

Previous international studies have consistently shown that therapeutic adherence in patients with T2DM is a multifactorial phenomenon influenced by patient-related, disease-related, treatment-related, and healthcare system factors. In particular, greater treatment complexity, polypharmacy, and mental health disorders have been associated with poorer adherence, especially among older adults with chronic conditions (9–11, 26, 27). Compared with previous national and international studies (9, 29–35), this study provides additional evidence by focusing on a large cohort of high-risk patients with T2DM managed in routine primary care and identified through the AMG system. By incorporating multiple dimensions potentially associated with adherence, including sociodemographic, functional, clinical, lifestyle-related, pharmacological, and healthcare utilization characteristics, this study contributes to a more comprehensive understanding of therapeutic nonadherence in a population with particularly high clinical complexity and healthcare needs.

Future longitudinal studies should build on these findings by examining changes in medication adherence over time and evaluating how adherence is associated with treatment effectiveness and relevant clinical outcomes, including glycaemic control, complications, and healthcare utilization. Such studies would help clarify temporal relationships and further establish the clinical relevance of medication adherence in high-risk patients with T2DM.

### 4.3 Applicability and implications for practice

These findings have several implications for primary care. First, therapeutic adherence should be routinely assessed in high-risk patients with T2DM, particularly those presenting with polypharmacy, complex multimorbidity, mental health conditions, respiratory or cardiovascular diseases, palliative care needs, or frequent healthcare utilization. The identification of these characteristics may help clinicians prioritize patients who require more detailed adherence assessments.

Second, interventions to improve adherence should extend beyond patient education alone. The association between nonadherence and medication burden highlights the importance of structured medication review, identification of potentially inappropriate or avoidable treatment complexity, simplification of therapeutic regimens when clinically appropriate, and shared decision-making (27). These strategies are particularly relevant in older adults with multimorbidity, where treatment goals should be individualized according to clinical priorities, functional status, and patient preferences.

Third, primary care teams are well positioned to implement multidisciplinary adherence strategies. Family physicians, nurses, and pharmacists can contribute through medication reconciliation, assessment of treatment burden, monitoring of adverse drug reactions, reinforcement of self-management strategies, and structured follow-up. The higher healthcare utilization observed among nonadherent patients suggests that routine primary care contacts may represent valuable opportunities to detect adherence difficulties and implement targeted interventions rather than requiring separate adherence-focused encounters.

Finally, AMG-based risk stratification may support the identification of patients with greater vulnerability to therapeutic nonadherence. Integrating clinical complexity assessment with information on medication burden, specific comorbidities, behavioral factors, and healthcare utilization may facilitate a more targeted allocation of adherence interventions within primary care.

### 4.4 Strengths and limitations

A major strength of this study is the inclusion of a large real-world cohort of high-risk patients with T2DM managed in primary care, providing relevant evidence on therapeutic adherence in a population with substantial clinical complexity and high healthcare needs. The use of routinely collected electronic health records linked to pharmacy dispensing data enabled the evaluation of multiple domains potentially associated with adherence, including sociodemographic, functional, clinical, lifestyle-related, pharmacological, and healthcare utilization characteristics. Furthermore, the use of the AMG stratification system enhances the clinical relevance of the findings by allowing the identification of patients with greater complexity who may benefit from adherence-focused interventions.

Several limitations should be acknowledged. First, the cross-sectional design limits the ability to establish temporal relationships and causality; therefore, associations should be interpreted as potential determinants or markers of nonadherence. Second, adherence was assessed using pharmacy dispensing records, which provide an objective measure of medication acquisition but cannot confirm actual medication intake. In addition, the PDC calculation was based on the minimum recommended daily dose for each medication. This assumption may overestimate medication coverage when patients receive or use higher doses and may consequently result in some misclassification of adherence. This potential limitation should be considered when interpreting the adherence estimates. Third, patients without available adherence information were excluded from the comparative analyses, which may have introduced some selection bias. In addition, some lifestyle-related variables had substantial proportions of missing data and should therefore be interpreted with caution. Although the IHC categories provided information related to healthcare coverage and pharmaceutical contribution levels, they should not be considered a comprehensive measure of socioeconomic status. Finally, the large sample size may have increased the likelihood of detecting statistically significant differences of limited clinical magnitude. This should be considered alongside the relatively low explanatory performance of the final model, indicating that the identified factors explain only a modest proportion of the variability in nonadherence. However, this may be expected for a complex, multidimensional behavior such as medication adherence, which is influenced by a wide range of related factors. Therefore, odds ratios close to 1, although statistically significant, should be interpreted cautiously in terms of their practical or clinical relevance. Furthermore, the findings are derived from a large public primary care system, their generalizability to other healthcare settings should be considered according to differences in healthcare organization and population characteristics.

## 5 Conclusions

Therapeutic nonadherence was highly prevalent among high-risk patients with T2DM managed in routine primary care. Nonadherence was associated with greater clinical complexity, higher treatment burden, selected cardiovascular, respiratory, and mental health conditions, smoking status, palliative care use, and increased healthcare utilization among a universally multimorbid population. Although sociodemographic differences were generally limited, some demographic and social factors remained independently associated with nonadherence after adjustment.

These findings indicate that therapeutic nonadherence in high-risk patients with T2DM is closely related to the complexity of chronic disease management, including treatment burden, specific comorbidity patterns, behavioral factors, and healthcare needs. Routine adherence assessment, medication review, and individualized care strategies may help primary care teams identify patients who require additional support and optimize diabetes management. Future longitudinal studies are warranted to confirm these associations and evaluate interventions aimed at improving therapeutic adherence in this high-risk population.

## Data Availability

The datasets generated and analyzed during the current study are not publicly available because they belong to the Madrid Health Service but are available from the corresponding author upon reasonable request. Requests to access the datasets should be directed to jaime.barrio@salud.madrid.org.

## Supplementary material

Supplementary Table 1 Study variables and their definitions/measurements.

Supplementary Table 2 Type of chronic diseases among high-risk patients with T2DM overall and by therapeutic adherence status.

Supplementary Figure 1 Flowchart of study population selection.

## Glossary

ADA: American Diabetes Association
ADHD: attention-deficit/hyperactivity disorder
AMG: Adjusted Morbidity Groups
ATC: Anatomical Therapeutic Chemical
BEAMER: BEhavioral and Adherence Model for improving quality, health outcomes, and cost-Effectiveness of healthcaRe
CNS: central nervous system
CI: Confidence intervals
COPD: chronic obstructive pulmonary disease
EASD: European Association for the Study of Diabetes
EFPIA: European Federation of Pharmaceutical Industries and Associations
FIBHUNJ: Foundation for Biomedical Research of Hospital Niño Jesús
FIIBAP: Foundation for Biosanitary Research and Innovation in Primary Care
HIV: human immunodeficiency virus
IHC: Individual Health Card
IMI: Innovative Medicines Initiative
IQR: interquartile range
OR: odds ratios
P: p-value
PDC: Proportion of days covered
Q: Quartile
R^2^: Coefficient of determination
RICAPPS: Research Network on Chronicity, Primary Health Care and Prevention and Health Promotion
SERMAS: Madrid Health Service
SD: standard deviation
SPSS: Statistical Package for social sciences
T2DM: Type 2 diabetes mellitus
UCJC: University Camilo José
