# Supplementary material for: Therapeutic adherence and associated factors among high-risk patients with type 2 diabetes in primary healthcare: a cross-sectional study using real-world data from the Madrid Region
Source: Front Endocrinol (Lausanne). 2026 Sep 7;17:1947294. doi: 10.3389/fendo.2026.1947294 (PMC13590293; doi:10.3389/fendo.2026.1947294)
Supplement: Supplementary file 1 [file SupplementaryFile1.docx]

**Supplementary Figure 1. Flowchart of study population selection.**

AMG: adjusted morbidity groups; HER, electronic health records; PDC, Proportion of days covered; T2DM, type 2 diabetes mellitus.
