# Supplementary material for: Therapeutic adherence and associated factors among high-risk patients with type 2 diabetes in primary healthcare: a cross-sectional study using real-world data from the Madrid Region
Source: Front Endocrinol (Lausanne). 2026 Sep 7;17:1947294. doi: 10.3389/fendo.2026.1947294 (PMC13590293; doi:10.3389/fendo.2026.1947294)
Supplement: Supplementary file 2 [file Table1.docx]

**Supplementary Table 1**. **Study variables and their definitions/measurements**

| **Variable Category** | **Variable** | **Definition/Measurement** |
| --- | --- | --- |
| **Sociodemographic** | Age | Age in years |
|  | Sex | Male/Female |
|  | Foreign status | Yes/No |
|  | Continent of birth | Europe/America/Africa/Asia/Oceania |
|  | Deprivation index | Composite socioeconomic deprivation index, analysed as a continuous variable and categorized into quartiles according to its distribution:  Quartile 1 (Q1) included values ≤ the 25th percentile, representing the less deprived group  Quartile 2 (Q2) included values > P25 and ≤ the 50th percentile  Quartile 3 (Q3) included values > P50 and ≤ the 75th percentile  Quartile 4 (Q4) included values > P75 up to the maximum value, representing the most deprived group |
|  | Individual Health Card income category (IHC) | Administrative variable linked to income level, employment status, and special conditions:  IHC 001 = Exempt users, noncontributory pensioners, and long-term unemployed individuals  IHC 002 = Pensioners with a 10% copayment  IHC 003 = Active citizens with income up to €18,000, copayment 40%  IHC 004 = Active citizens with income between €18,000–€100,000, copayment 50%  IHC 005 = Active and passive citizens with income above €100,000, copayment 60%  IHC 006 = Citizens belonging to mutual insurance schemes  Other IHC categories = categories not included in the main categories analyzed. |
| **Functional** | Dependency | Yes/No |
|  | Living alone | Yes/No |
|  | Homebound/immobility | Yes/No |
|  | Primary caregiver | Children/Partner/Other relatives/Home care/Other/Parents |
|  | Palliative care | Yes/No |
| **Clinical** | AMG index | Composite morbidity-based complexity index, analysed as a numerical variable |
|  | Number of chronic   diseases | Count of chronic conditions, analysed as a numerical variable |
|  | Multimorbidity | Presence of ≥ 2 chronic diseases |
|  | Chronic disease categories | Cardiovascular, Digestive/Hepatic, Endocrine/Metabolic, Eye, Genitourinary, Infectious diseases, Mental and behavioural disorders, Musculoskeletal/Connective tissue, Neurological, Oncological, Respiratory |
|  | Type of disease | ADHD, Neoplasm, Alcohol abuse disorder, Anaemia, Anxiety, Arthritis, Asthma, Aortic aneurysm, Autism, Bladder cancer, Bipolar disorder, Breast cancer, Central Nervous System cancer, Cardiac conduction disorder, Cardiomyopathy, Cardiopulmonary disease, Chronic heart failure, Chronic kidney disease, Chronic pain, Chronic Pancreatitis, Cirrhosis, Colon cancer, COPD, Celiac disease, Dementia, Depression, Disc disease, Dysrhythmias, Epilepsy, Hepatic disease, Gastrointestinal haemorrhage, Glaucoma, Hodgkin lymphoma, Hyperlipidaemia, Intellectual disability, Inflammatory bowel disease, Ischaemic heart disease, Kidney cancer, Leukaemia, Lupus, Lung cancer, Melanoma, Multiple myeloma, Multiple sclerosis, Obesity, Obsessive-compulsive disorder, Osteoarthritis, Osteoporosis, Pancreatic cancer, Parkinson’s disease, Peptic ulcer disease, Prostate cancer, Psychotic disorder, Recurrent urinary tract infections, Respiratory diseases, Spinal cord injury, Stomach cancer, Stroke, Substance abuse disorder, Thyroid cancer, Thyroid disorder, Tuberculosis, Ulcerative colitis/Crohn’s disease, Valvular disease |
| **Lifestyles - related** | Tobacco use | Yes/No |
|  | Alcohol abuse | Yes/No |
|  | Illicit substance abuse | Yes/No |
|  | Medication abuse | Yes/No |
|  | Mediterranean diet | Yes/No |
|  | Physical exercise | Yes/No |
|  | Normal sleep habits | Categorical variable according to electronic health record coding |
|  | Regular stress | Categorical variable according to electronic health record coding |
|  | Risk of sexually transmitted infections | Yes/No |
| **Pharmacological** | Number of medications | Count of dispensed medications, analysed as a numerical variable |
|  | Polypharmacy | Use of ≥ 5 medications |
|  | Therapeutic adherence | Proportion of days covered (PDC) ≥ 80% during a 12-month period |
|  | Concern about medication | Yes/No |
|  | Adverse drug reactions | Yes/No |
|  | Treatment complications | Yes/No |
| **Primary care healthcare services** | Total contacts | Number of primary care contacts, analysed as a numerical variable |
|  | Professional contacted | Number of contacts with a family medicine doctor, primary care nurse and primary care pharmacist (numerical value) |
|  | Mode of contact | Number of in–person consultations, phone, home visits (numerical value) |

Abbreviations: ADHD, attention-deficit/hyperactivity disorder; AMG, adjusted morbidity groups; COPD, chronic obstructive pulmonary disease; IHC, Individual Health Card income category; PDC, proportion of days covered; STI, sexually transmitted infections; T2DM, Type 2 diabetes mellitus.
