# Supplementary material for: Therapeutic adherence and associated factors among high-risk patients with type 2 diabetes in primary healthcare: a cross-sectional study using real-world data from the Madrid Region
Source: Front Endocrinol (Lausanne). 2026 Sep 7;17:1947294. doi: 10.3389/fendo.2026.1947294 (PMC13590293; doi:10.3389/fendo.2026.1947294)
Supplement: Supplementary file 3 [file Table2.docx]

**Supplementary Table 2. Type of chronic diseases among high-risk patients with T2DM overall and by therapeutic adherence status.**

| **Clinical Characteristics**  **n (%)** | **Patients with T2DM**  **n= 65,856 (100)** | **Nonadherent**  **n= 45,669 (69.3)** | **Adherent**  **n= 20,187 (30.7)** | **p value** |
| --- | --- | --- | --- | --- |
| **Cardiovascular diseases** | | | | |
| Aortic aneurysm | 984 (1.5) | 696 (1.5) | 288 (1.4) | 0.342 |
| Cardiac conduction disorder | 4,337 (6.6) | 2,976 (6.5) | 1,361 (6.7) | 0.282 |
| Cardiomyopathy | 2,000 (3.0) | 1,427 (3.1) | 573 (2.8) | 0.048 |
| Cardiopulmonary disease | 4,139 (6.3) | 2,956 (6.5) | 1,183 (5.9) | 0.003 |
| Chronic heart failure | 16,099 (24.4) | 11,502 (25.2) | 4,597 (22.8) | <0.001 |
| Dysrhythmias | 24,182 (36.7) | 16,817 (36.8) | 7,365 (36.5) | 0.404 |
| Hypertension | 58,283 (88.5) | 40,399 (88.5) | 17,884 (88.6) | 0.626 |
| Ischaemic heart disease | 19,250 (29.2) | 13,668 (29.9) | 5,582 (27.7) | <0.001 |
| Valvular disease | 6,548 (9.9) | 4,597 (10.1) | 1,951 (9.7) | 0.113 |
| **Digestive and hepatic diseases** | | | | |
| Celiac disease | 92 (0.1) | 66 (0.1) | 26 (0.1) | 0.618 |
| Cirrhosis | 467 (0.7) | 318 (0.7) | 149 (0.7) | 0.556 |
| Chronic pancreatitis | 267 (0.4) | 177 (0.4) | 90 (0.4) | 0.278 |
| Inflammatory bowel disease | 247 (0.4) | 171 (0.4) | 76 (0.4) | 0.968 |
| Fatty liver disease | 9,553 (14.5) | 6,734 (14.7) | 2,819 (14.0) | 0.009 |
| Peptic ulcer disease | 2,696 (4.1) | 1,873 (4.1) | 823 (4.1) | 0.884 |
| Ulcerative colitis/Crohn´s disease | 699 (1.1) | 486 (1.1) | 213 (1.1) | 0.917 |
| **Metabolic and Endocrine diseases** | | | | |
| Hyperlipidaemia | 48,749 (74.0) | 33,925 (74.3) | 14,824 (73.4) | 0.022 |
| Obesity | 24,510 (37.2) | 17,210 (37.7) | 7,300 (36.2) | <0.001 |
| Thyroid disorder | 18,400 (27.9) | 12,819 (28.1) | 5,581 (27.6) | 0.265 |
| **Eye diseases** | | | | |
| Glaucoma | 6,944 (10.5) | 4,760 (10.4) | 2,184 (10.8) | 0.127 |
| **Genitourinary diseases** | | | | |
| Chronic kidney disease | 15,726 (23.9) | 10,952 (24.0) | 4,774 (23.6) | 0.356 |
| **Haematologic disorders** | | | | |
| Anaemia | 22,067 (33.5) | 14,981 (32.8) | 7,086 (35.1) | <0.001 |
| **Infectious diseases** | | | | |
| Tuberculosis | 925 (1.4) | 647 (1.4) | 278 (1.4) | 0.691 |
| Recurrent urinary tract infections | 3,350 (5.1) | 2,278 (5.0) | 1,072 (5.3) | 0.083 |
| HIV | 426 (0.6) | 299 (0.7) | 127 (0.6) | 0.706 |
| **Mental and behavioural disorders** | | | | |
| ADHD | 147 (0.2) | 99 (0.2) | 48 (0.2) | 0.599 |
| Alcohol abuse disorder | 3,155 (4.8) | 2,245 (4.9) | 910 (4.5) | 0.024 |
| Anxiety | 2,674 (4.1) | 1,869 (4.1) | 805 (4.0) | 0.530 |
| Autism | 0 (0.0) | 0 (0.0) | 0 (0.0) | --- |
| Bipolar disorder | 482 (0.7) | 308 (0.7) | 174 (0.9) | 0.009 |
| Depression | 17,931 (27.2) | 12,599 (27.6) | 5,332 (26.4) | 0.002 |
| Intellectual disability | 10 (0.0) | 9 (0.0) | 1 (0.0) | 0.157 |
| Obsessive-compulsive disorder | 117 (0.2) | 94 (0.2) | 23 (0.1) | 0.010 |
| Psychotic disorder | 659 (1.0) | 427 (0.9) | 232 (1.1) | 0.011 |
| Substance abuse disorder | 483 (0.7) | 354 (0.8) | 129 (0.6) | 0.059 |
| **Musculoskeletal and connective tissue diseases** | | | | |
| Arthritis | 4,591 (7.0) | 3,228 (7.1) | 1,363 (6.8) | 0.142 |
| Lupus | 9 (0.0) | 8 (0.0) | 1 (0.0) | 0.203 |
| Chronic pain | 140 (0.2) | 93 (0.2) | 47 (0.2) | 0.453 |
| Osteoarthritis | 36,613 (55.6) | 25,487 (55.8) | 11,126 (55.1) | 0.099 |
| Osteoporosis | 9,243 (14.0) | 6,485 (14.2) | 2,758 (13.7) | 0.067 |
| **Neurological disorders** | | | | |
| Dementia | 6,915 (10.5) | 4,574 (10.0) | 2,341 (11.6) | <0.001 |
| Disc disease | 25,475 (38.7) | 17,786 (38.9) | 7,689 (38.1) | 0.037 |
| Epilepsy/seizure disorder | 1,781 (2.7) | 1,181 (2.6) | 600 (3.0) | 0.005 |
| Multiple sclerosis | 144 (0.2) | 93 (0.2) | 51 (0.3) | 0.215 |
| Paralysis | 837 (1.3) | 551 (1.2) | 286 (1.4) | 0.026 |
| Parkinson´s disease | 2,041 (3.1) | 1,368 (3.0) | 673 (3.3) | 0.021 |
| Spinal cord injury | 70 (0.1) | 51 (0.1) | 19 (0.1) | 0.524 |
| Stroke | 13,570 (20,6 ) | 9,329 (20.4) | 4,241 (21.0) | 0.089 |
| **Oncological diseases** | | | | |
| Active Neoplasm | 3,505 (5.3) | 2,436 (5.3) | 1,069 (5.3) | 0.839 |
| Bladder cancer | 1,216 (1.8) | 846 (1.9) | 370 (1.8) | 0.863 |
| Breast cancer | 455 (0.7) | 312 (0.7) | 143 (0.7) | 0.719 |
| CNS cancer | 29 (0.0) | 21 (0.0) | 8 (0.0) | 0.720 |
| Cervical cancer | 34 (0.1) | 22 (0.0) | 12 (0.1) | 0.557 |
| Colon cancer | 1,016 (1.5) | 663 (1.5) | 353 (1.7) | 0.004 |
| Hodgkin lymphoma/other lymphomas | 145 (0.2) | 102 (0.2) | 43 (0.2) | 0.794 |
| Kidney cancer | 212 (0.3) | 145 (0.3) | 67 (0.3) | 0.764 |
| Leukaemia | 213 (0.3) | 139 (0.3) | 74 (0.4) | 0.195 |
| Lung cancer | 432 (0.7) | 316 (0.7) | 116 (0.6) | 0.086 |
| Melanoma | 101 (0.2) | 72 (0.2) | 29 (0.1) | 0.672 |
| Multiple myeloma | 132 (0.2) | 86 (0.2) | 46 (0.2) | 0.295 |
| Prostate cancer | 854 (1.3) | 570 (1.2) | 284 (1.4) | 0.097 |
| Pancreatic cancer | 108 (0.2) | 68 (0.1) | 40 (0.2) | 0.150 |
| Stomach cancer | 140 (0.2) | 96 (0.2) | 44 (0.2) | 0.842 |
| Thyroid cancer | 118 (0.2) | 77 (0.2) | 41 (0.2) | 0.334 |
| **Respiratory diseases** |  |  |  |  |
| Asthma | 11,308 (17.2) | 9,066 (19.9) | 2,242 (11.1) | <0.001 |
| Cystic fibrosis | 11 (0.0) | 6 (0.0) | 5 (0.0) | 0.287 |
| COPD | 12,004 (18.2) | 9,531 (20.9) | 2,473 (12.3) | <0.001 |

Abbreviations: ADHD, attention-deficit/hyperactivity disorder; CNS, central nervous system; COPD, chronic obstructive pulmonary disease; HIV, human immunodeficiency virus; T2DM, type 2 diabetes mellitus. Patients with unavailable therapeutic adherence data were excluded from the analyses (n = 1,890; 2.8%).
